# Poly[tetra­methyl­ammonium [tri-μ_2_-formato-κ^6^ *O*:*O*′-manganate(II)]]

**DOI:** 10.1107/S1600536813024045

**Published:** 2013-09-12

**Authors:** Cai-Yun Han, Min-Min Liu, Qin-Qin Dang

**Affiliations:** a School of Chemistry and Material Science, Shanxi Normal University, Linfen 041004, People’s Republic of China

## Abstract

In the title compound, {(C_4_H_12_N)[Mn(HCO_2_)_3_]}_*n*_, the Mn^II^ atom lies on an inversion centre and is coordinated by O-atom donors from the three double-bridging formate ligands, one of which lies across a crystallographic mirror plane, giving a slightly distorted octahedral coordination sphere. A three-dimensional NaCl-type framework is generated in which the tetra­methyl­ammonium cations, which lie across mirror planes and occupy the cavities in the polymer structure, form weak C—H⋯O hydrogen bonds with the formate ligands.

## Related literature

For related structures, see: Gao & Ng (2010 ▶); Wang *et al.* (2004 ▶, 2010 ▶). For background to the properties of structures with metal–formate frameworks templated by protonated amines, see: Liu *et al.* (2012 ▶); Zhang *et al.* (2007 ▶).
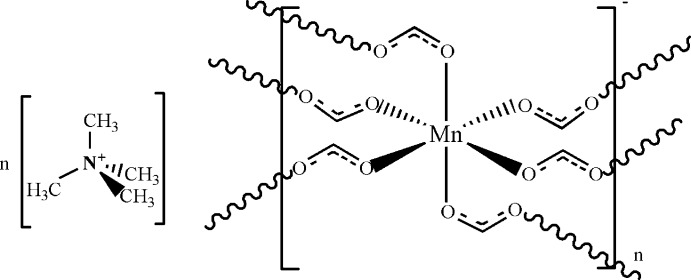


## Experimental

### 

#### Crystal data

(C_4_H_12_N)[Mn(HCO_2_)_3_]
*M* *_r_* = 264.14
Orthorhombic, 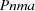

*a* = 8.926 (4) Å
*b* = 12.767 (6) Å
*c* = 9.196 (4) Å
*V* = 1048.0 (8) Å^3^
*Z* = 4
Mo *K*α radiation
μ = 1.27 mm^−1^
*T* = 298 K
0.22 × 0.22 × 0.15 mm

#### Data collection

Bruker SMART CCD area-detector diffractometer
Absorption correction: multi-scan (*SADABS*; Bruker, 2007 ▶) *T*_min_ = 0.768, *T*_max_ = 0.833
5907 measured reflections
1189 independent reflections
1012 reflections with *I* > 2σ(*I*)
*R*_int_ = 0.023

#### Refinement

*R*[*F* ^2^ > 2σ(*F* ^2^)] = 0.027
*wR*(*F* ^2^) = 0.075
*S* = 0.98
1189 reflections
77 parameters
H-atom parameters constrained
Δρ_max_ = 0.27 e Å^−3^
Δρ_min_ = −0.43 e Å^−3^

### 

Data collection: *SMART* (Bruker, 2007 ▶); cell refinement: *SAINT-Plus* (Bruker, 2007 ▶); data reduction: *SAINT-Plus*; program(s) used to solve structure: *SHELXS97* (Sheldrick, 2008 ▶); program(s) used to refine structure: *SHELXL97* (Sheldrick, 2008 ▶); molecular graphics: *DIAMOND* (Brandenburg & Putz, 2006 ▶); software used to prepare material for publication: *SHELXTL* (Sheldrick, 2008 ▶).

## Supplementary Material

Crystal structure: contains datablock(s) I, New_Global_Publ_Block. DOI: 10.1107/S1600536813024045/zs2272sup1.cif

Structure factors: contains datablock(s) I. DOI: 10.1107/S1600536813024045/zs2272Isup2.hkl

Additional supplementary materials:  crystallographic information; 3D view; checkCIF report

## Figures and Tables

**Table 1:** Selected bond lengths (Å)

Mn1—O1	2.2176 (16)
Mn1—O2	2.2010 (15)
Mn1—O3^i^	2.2089 (16)
Mn1—O1^ii^	2.2176 (16)
Mn1—O2^ii^	2.2010 (15)
Mn1—O3^iii^	2.2089 (16)

Symmetry codes: (i) 
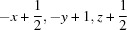
; (ii) 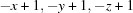; (iii) 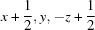.

**Table 2:** Hydrogen-bond geometry (Å, °)

*D*—H⋯*A*	*D*—H	H⋯*A*	*D*⋯*A*	*D*—H⋯*A*
C3—H3*B*⋯O3^ii^	0.96	2.40	3.355 (3)	176
C4—H4*B*⋯O2^iv^	0.96	2.41	3.367 (3)	171
C5—H5*B*⋯O1^iii^	0.96	2.41	3.349 (3)	167

Symmetry codes: (ii) 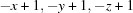; (iii) 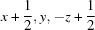; (iv) 
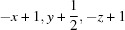
.

**Fig. 1.**
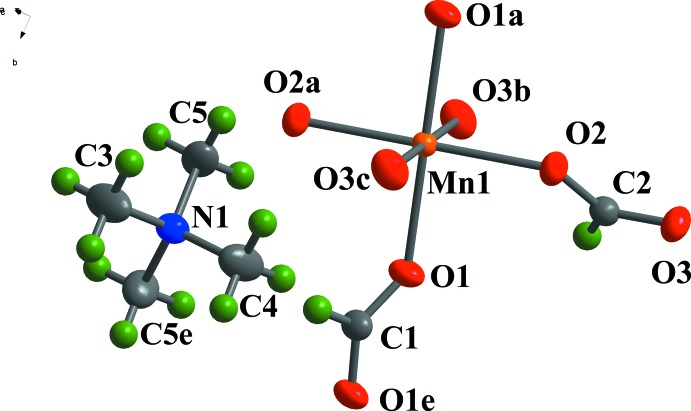
Atom numbering scheme for the cation and anion species in the title complex, with displacement ellipsoids drawn at the 50% probability level. For symmetry codes: (a) -x +1, -y +1, -z +1; (b) x +1/2, y, -z +1/2; (c) -x +1/2, -y +1, z +1/2; (e) x, -y +3/2, z.

**Fig. 2.**
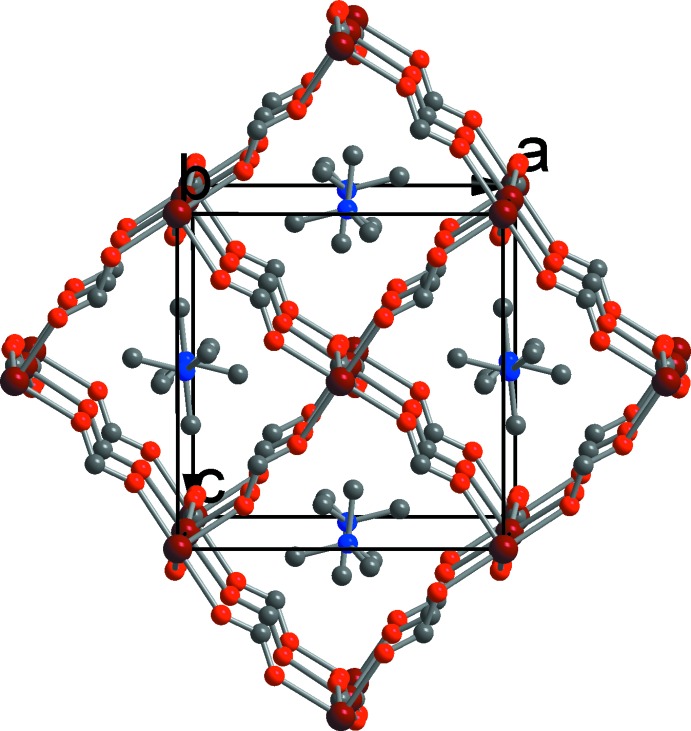
A view of the three-dimensional NaCl-like structure of the title compound

## supplementary crystallographic information

### 1. Comment

Recently, metal formate frameworks templated by protonated amines attract considerable attention because their special structures and interesting properties (Liu *et al.*, 2012; Zhang *et al.*, 2007). A series of protonated ammonium cations (methylamine, ethylamine, dimethylamine, cyclotrimethyleneamine, *N*,*N*-dimethylethylenediammonium) have been employed to synthesize metal formate frameworks (Gao *et al.*, 2010; Wang *et al.*, 2010; Wang *et al.*, 2004). In the present contribution, we report a new manganese formate complex {(Me_4_N) [Mn(HCO_2_)]_3_}_n_, templated using tetramethylamine cations. The formate ligand and tetramethylamine cation result from an *in situ* decomposition reaction of the *N*,*N*-dimethylformamide solvent.

In the title complex (Fig. 1), the Mn^II^ cations lie on crystallographic inversion centres and are coordinated by six O-atom donors from bridging formate ligands, one of which lies across a crystallographic mirror plane and gives an *anti-anti* mode. A slightly distorted octahedral geometry is found about Mn^II^[Mn1—O1, Mn1—O2 and Mn1—O3 are 2.2176 (16), 2.2010 (15) and 2.2089 (16) Å, respectively (Table 1)] while the Mn—Mn separations across the bridging formates are 6.3835 (20)–6.4078 (20) Å. A three-dimensional NaCl-type framework is generated in which the protonated tetramethylamine cations, which also lie on crystallographic mirror planes, occupy the polymer cavities, acting as templates for charge-compensation and space-filling (Fig.2) and are weakly associated with the formate cations through C—H···O hydrogen bonds (Table 2).

### 2. Experimental

The title compound compound was synthesized hydrothermally under autogenous pressure. Typically, a mixture of manganese(II) acetate (0.049 g, 0.2 mmol), sodium hydroxide(0.008 g, 0.2 mmol), *N*,*N*-dimethylformamide (4 ml) and methanol (2.5 ml) was loaded into a 15 ml Teflon-lined stainless container and stirred in air for 20 minutes, then heated to 180 °C for 5 days. After cooling to room temperature and filtering, colorless block crystals were recovered in 90% yield.

### 3. Refinement

Hydrogen atoms of the organic groups were placed at calculated positions with C—H = 0.96 Å (methyl) or C—H = 0.93 Å (formyl) and allowed to ride, with *U*_iso_(H) = 1.2 or 1.5*U*_eq_(C).

### Crystal data

**Table:**

(C_4_H_12_N)[Mn(HCO_2_)_3_]	*F*(000) = 548
*M**_r_* = 264.14	*D*_x_ = 1.674 Mg m^−^^3^
Orthorhombic, *P**n**m**a*	Mo *K*α radiation, λ = 0.71073 Å
Hall symbol: -P 2ac 2n	Cell parameters from 2391 reflections
*a* = 8.926 (4) Å	θ = 3.2–28.6°
*b* = 12.767 (6) Å	µ = 1.27 mm^−^^1^
*c* = 9.196 (4) Å	*T* = 298 K
*V* = 1048.0 (8) Å^3^	Block, colorless
*Z* = 4	0.22 × 0.22 × 0.15 mm

### Data collection

**Table:**

Bruker SMART CCD area-detector diffractometer	1189 independent reflections
Radiation source: fine-focus sealed tube	1012 reflections with *I* > 2σ(*I*)
Graphite monochromator	*R*_int_ = 0.023
Φ and ω scans	θ_max_ = 27.0°, θ_min_ = 2.7°
Absorption correction: multi-scan (*SADABS*; Bruker, 2007)	*h* = −11→8
*T*_min_ = 0.768, *T*_max_ = 0.833	*k* = −16→16
5907 measured reflections	*l* = −11→11

### Refinement

**Table:**

Refinement on *F*^2^	Primary atom site location: structure-invariant direct methods
Least-squares matrix: full	Secondary atom site location: difference Fourier map
*R*[*F*^2^ > 2σ(*F*^2^)] = 0.027	Hydrogen site location: inferred from neighbouring sites
*wR*(*F*^2^) = 0.075	H-atom parameters constrained
*S* = 0.98	*w* = 1/[σ^2^(*F*_o_^2^) + (0.041*P*)^2^ + 0.4246*P*] where *P* = (*F*_o_^2^ + 2*F*_c_^2^)/3
1189 reflections	(Δ/σ)_max_ < 0.001
77 parameters	Δρ_max_ = 0.27 e Å^−^^3^
0 restraints	Δρ_min_ = −0.43 e Å^−^^3^

### Special details

**Table:**

Geometry. Bond distances, angles etc. have been calculated using the rounded fractional coordinates. All su's are estimated from the variances of the (full) variance-covariance matrix. The cell esds are taken into account in the estimation of distances, angles and torsion angles
Refinement. Refinement of *F*^2^ against ALL reflections. The weighted *R*-factor *wR* and goodness of fit *S* are based on *F*^2^, conventional *R*-factors *R* are based on *F*, with *F* set to zero for negative *F*^2^. The threshold expression of *F*^2^ > σ(*F*^2^) is used only for calculating *R*-factors(gt) *etc*. and is not relevant to the choice of reflections for refinement. *R*-factors based on *F*^2^ are statistically about twice as large as those based on *F*, and *R*- factors based on ALL data will be even larger.

### Fractional atomic coordinates and isotropic or equivalent isotropic displacement parameters (Å^2^)

**Table:**

	*x*	*y*	*z*	*U*_iso_*/*U*_eq_	
Mn1	0.50000	0.50000	0.50000	0.0196 (1)	
O1	0.48275 (14)	0.66330 (9)	0.41954 (13)	0.0353 (4)	
O2	0.29850 (13)	0.45825 (9)	0.37482 (12)	0.0328 (3)	
O3	0.13485 (14)	0.45849 (10)	0.19299 (12)	0.0370 (4)	
C1	0.4883 (3)	0.75000	0.4754 (3)	0.0294 (7)	
C2	0.25485 (17)	0.48251 (13)	0.25112 (16)	0.0264 (5)	
N1	1.0114 (2)	0.75000	0.4921 (2)	0.0310 (7)	
C3	1.0293 (4)	0.75000	0.6524 (3)	0.0565 (12)	
C4	0.8494 (3)	0.75000	0.4525 (4)	0.0515 (10)	
C5	1.0830 (2)	0.65471 (15)	0.4312 (2)	0.0461 (6)	
H1	0.49830	0.75000	0.57610	0.0350*	
H2	0.31980	0.52340	0.19600	0.0320*	
H3A	1.13400	0.75000	0.67640	0.0680*	
H3B	0.98280	0.68860	0.69230	0.0680*	
H4A	0.83940	0.75000	0.34860	0.0620*	
H4B	0.80230	0.81140	0.49170	0.0620*	
H5A	1.18770	0.65480	0.45470	0.0690*	
H5B	1.07080	0.65410	0.32740	0.0690*	
H5C	1.03660	0.59360	0.47200	0.0690*	

### Atomic displacement parameters (Å^2^)

**Table:**

	*U*^11^	*U*^22^	*U*^33^	*U*^12^	*U*^13^	*U*^23^
Mn1	0.0205 (2)	0.0185 (2)	0.0197 (2)	−0.0002 (1)	−0.0002 (1)	0.0001 (1)
O1	0.0513 (8)	0.0212 (6)	0.0335 (7)	0.0006 (5)	−0.0021 (5)	0.0020 (5)
O2	0.0316 (6)	0.0361 (6)	0.0306 (6)	−0.0047 (5)	−0.0094 (5)	0.0038 (5)
O3	0.0361 (7)	0.0417 (7)	0.0332 (6)	−0.0044 (6)	−0.0128 (5)	0.0073 (5)
C1	0.0396 (15)	0.0277 (13)	0.0210 (10)	0.0000	−0.0014 (9)	0.0000
C2	0.0277 (9)	0.0245 (7)	0.0271 (9)	−0.0021 (6)	0.0012 (7)	0.0010 (6)
N1	0.0359 (13)	0.0271 (12)	0.0301 (12)	0.0000	0.0039 (8)	0.0000
C3	0.103 (3)	0.0385 (16)	0.0279 (14)	0.0000	0.0075 (15)	0.0000
C4	0.0307 (14)	0.0389 (15)	0.085 (2)	0.0000	0.0086 (15)	0.0000
C5	0.0478 (11)	0.0430 (11)	0.0474 (11)	0.0158 (9)	−0.0043 (9)	−0.0143 (9)

### Geometric parameters (Å, º)

**Table:**

Mn1—O1	2.2176 (16)	N1—C5^iv^	1.484 (2)
Mn1—O2	2.2010 (15)	C1—H1	0.9300
Mn1—O3^i^	2.2089 (16)	C2—H2	0.9300
Mn1—O1^ii^	2.2176 (16)	C3—H3A	0.9600
Mn1—O2^ii^	2.2010 (15)	C3—H3B	0.9600
Mn1—O3^iii^	2.2089 (16)	C3—H3B^iv^	0.9600
O1—C1	1.2213 (17)	C4—H4A	0.9600
O2—C2	1.2417 (19)	C4—H4B	0.9600
O3—C2	1.236 (2)	C4—H4B^iv^	0.9600
N1—C4	1.491 (3)	C5—H5A	0.9600
N1—C5	1.484 (2)	C5—H5B	0.9600
N1—C3	1.483 (3)	C5—H5C	0.9600
			
O1—Mn1—O2	89.80 (4)	O1—C1—O1^iv^	130.0 (2)
O1—Mn1—O3^i^	90.27 (5)	O2—C2—O3	127.33 (15)
O1—Mn1—O1^ii^	180.00	O1—C1—H1	115.00
O1—Mn1—O2^ii^	90.20 (4)	O1^iv^—C1—H1	115.00
O1—Mn1—O3^iii^	89.73 (5)	O3—C2—H2	116.00
O2—Mn1—O3^i^	91.89 (4)	O2—C2—H2	116.00
O1^ii^—Mn1—O2	90.20 (4)	N1—C3—H3A	109.00
O2—Mn1—O2^ii^	180.00	N1—C3—H3B	109.00
O2—Mn1—O3^iii^	88.11 (4)	N1—C3—H3B^iv^	109.00
O1^ii^—Mn1—O3^i^	89.73 (5)	H3A—C3—H3B	109.00
O2^ii^—Mn1—O3^i^	88.11 (4)	H3A—C3—H3B^iv^	109.00
O3^i^—Mn1—O3^iii^	180.00	H3B—C3—H3B^iv^	110.00
O1^ii^—Mn1—O2^ii^	89.80 (4)	N1—C4—H4A	109.00
O1^ii^—Mn1—O3^iii^	90.27 (5)	N1—C4—H4B	109.00
O2^ii^—Mn1—O3^iii^	91.89 (4)	N1—C4—H4B^iv^	109.00
Mn1—O1—C1	135.15 (14)	H4A—C4—H4B	109.00
Mn1—O2—C2	132.47 (11)	H4A—C4—H4B^iv^	109.00
Mn1^v^—O3—C2	139.49 (11)	H4B—C4—H4B^iv^	110.00
C3—N1—C5	109.20 (13)	N1—C5—H5A	109.00
C3—N1—C5^iv^	109.20 (13)	N1—C5—H5B	109.00
C4—N1—C5	108.99 (13)	N1—C5—H5C	109.00
C4—N1—C5^iv^	108.99 (13)	H5A—C5—H5B	110.00
C5—N1—C5^iv^	110.13 (15)	H5A—C5—H5C	109.00
C3—N1—C4	110.3 (2)	H5B—C5—H5C	109.00
			
O2—Mn1—O1—C1	132.4 (2)	O1^ii^—Mn1—O2—C2	−139.34 (14)
O3^i^—Mn1—O1—C1	40.5 (2)	O3^iii^—Mn1—O2—C2	−49.08 (14)
O2^ii^—Mn1—O1—C1	−47.7 (2)	Mn1—O1—C1—O1^iv^	176.99 (17)
O3^iii^—Mn1—O1—C1	−139.5 (2)	Mn1—O2—C2—O3	−176.16 (12)
O1—Mn1—O2—C2	40.66 (14)	Mn1^v^—O3—C2—O2	168.34 (12)
O3^i^—Mn1—O2—C2	130.92 (14)		

Symmetry codes: (i) −*x*+1/2, −*y*+1, *z*+1/2; (ii) −*x*+1, −*y*+1, −*z*+1; (iii) *x*+1/2, *y*, −*z*+1/2; (iv) *x*, −*y*+3/2, *z*; (v) −*x*+1/2, −*y*+1, *z*−1/2.

### Hydrogen-bond geometry (Å, º)

**Table:**

*D*—H···*A*	*D*—H	H···*A*	*D*···*A*	*D*—H···*A*
C3—H3*B*···O3^ii^	0.96	2.40	3.355 (3)	176
C4—H4*B*···O2^vi^	0.96	2.41	3.367 (3)	171
C5—H5*B*···O1^iii^	0.96	2.41	3.349 (3)	167

Symmetry codes: (ii) −*x*+1, −*y*+1, −*z*+1; (iii) *x*+1/2, *y*, −*z*+1/2; (vi) −*x*+1, *y*+1/2, −*z*+1.
